# Direct transarterial embolization via the deep circumflex iliac artery for a type II endoleak using a novel Onyx™ mixture

**DOI:** 10.1093/jscr/rjaa526

**Published:** 2020-12-24

**Authors:** Paul Ghaly, Glen Schlaphoff, Jim Iliopoulos, Mehtab Ahmad

**Affiliations:** Vascular Surgery Department, Liverpool Hospital, SWSLHD, NSW 2173, Australia; Department of Interventional Radiologist, Liverpool Hospital, SWSLHD, NSW, 2173, Australia; Vascular Surgery Department, Liverpool Hospital, SWSLHD, NSW 2173, Australia; Vascular Surgery Department, Liverpool Hospital, SWSLHD, NSW 2173, Australia

## Abstract

Internal iliac artery aneurysms (IIAA) can be associated with abdominal aortic aneurysms. We describe a technique of successful transarterial embolization using a mixture of Onyx™ formulations in a 72-year-old with previous open and endovascular aneurysm repairs of his abdominal aorta and a residual large left IIAA causing a Type II endoleak. We demonstrate that utilization of the deep circumflex iliac artery is a safe and viable alternate route to treating IIAA when direct access is not achievable.

## INTRODUCTION

Type II endoleaks (T2E) are the most common complication after endovascular aortic aneurysm repair (EVAR) with transarterial, or direct sac puncture techniques increasingly being favoured over open surgical repair due to the challenging nature open surgery presents in accessing the feeding vessel(s) [1, 2]. Internal iliac artery aneurysms (IIAA) are found in 20% of abdominal aortic aneurysm (AAA) cases, with a high associated mortality rate in the event of rupture [3, 4].

Coil embolization, endovascular stent-graft placement or a combination of these treatment modalities have been described in the management of IIAA, however such procedures are complex and challenging, particularly in patients with tortuous iliac anatomy.

We describe a case of IIAA embolization using a novel Onyx™ liquid embolic solution (LES) (Medtronic, Minnesota, USA) mixture through an alternative transarterial route in a 72-year-old male with a large IIAA.

## CASE REPORT

A 72-year-old male with an extensive history of complicated aortic aneurysm interventions presented with urinary retention. His other co-morbidities included type 2 diabetes mellitus, chronic kidney disease, ischemic heart disease, hypertension and hyperlipidemia.

Aged 52, he had undergone uncomplicated treatment of a 60-mm infra-renal AAA via open surgical tube-graft repair at another center. Post-operative records indicate serial imaging demonstrating no aneurysmal extension over the subsequent 5 years, prior to him being lost to follow up. Fifteen years later, he presented with acute abdominal pain and CT angiography (CTA) demonstrated a 56-mm proximal anastomotic pseudoaneurysm with associated stranding and a left 58-mm saccular IIAA. His symptomatic abdominal aortic pseudoaneurysm was managed emergently with a Zenith TX2 36–77 (Cook Medical, Inc) endovascular proximal extension cuff, placed infra-renally via bilateral groin exposures. A type III endoleak from the cuff on post-operative imaging necessitated further intervention. A bifurcated endograft, with bilateral on-table internal iliac artery (IIA) embolizations and limb-extensions into both external iliac arteries (EIA) was planned.

Intra-operative attempts to embolise the left IIAA were abandoned due to an inability to track a sheath or catheters into the tortuous IIA. The right IIA was successfully embolized with a 16F Amplatzer™ vascular plug (Abbott, Golden Valley, MN). The endograft main body (Xenith™ 36–128) was introduced from the right groin, with limb extensions into both EIA (ZIMB™ 13–107 and 20–90). Completion angiogram runs confirmed patency of both renal arteries, occlusion of the right IIA with the left IIAA demonstrating slow, delayed filling. Consensus was that this T2E was secondary to pelvic collateral flow which would occlude with time.

**Figure 1:**
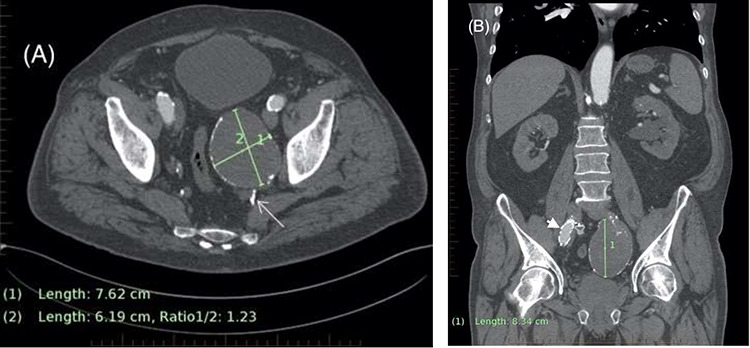
(**A**) Axial CT demonstrating left internal iliac aneurysm (IIAA) measuring ×76 62 mm with retrograde flow into the aneurysm (arrow) from the deep pelvic vessels. (**B**) Coronal CT demonstrating the left IIA with a craniocaudal length of 83 mm. The right external iliac artery endograft limb is also visible (arrowhead).

**Figure 2:**
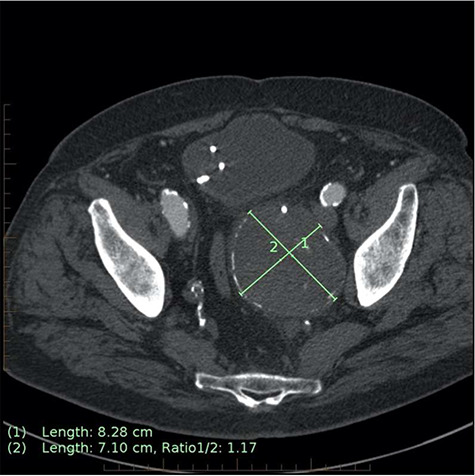
Axial CT image on 6-month follow-up demonstrating expansion of the IIAA by 7 mm, now measuring 83 × 71 mm axially (previously 76 × 62 mm).

After relocating to the catchment area of our hospital, he presented in acute urinary retention. A CT kidney, ureters and bladder (CTKUB) demonstrated left hydronephrosis and hydroureter secondary to extrinsic compression from the enlarged left IIAA (from 58 mm to 76 mm in diameter) (Fig. 1A and B). Embolization was delayed by the associated acute kidney injury, which was first managed by the urologists with J-J stent insertion for ureteric decompression. He then entered a period of repeated urological interventions due to complications from his ureteric instrumentation, including urosepsis requiring a prolonged intensive care unit admission. When his urinary tract infections had cleared, a CTA demonstrated the IIAA to have further increased (83 mm × 71 mm), prompting urgent embolization (Fig. 2).

The IIAA feeding vessels were traced to retrograde flow from the deep branches of the pelvis. An anastomosis from the left deep circumflex iliac artery (DCIA) was mapped as an access vessel to the IIAA. Under local anaesthesia, retrograde transarterial access was achieved through the left common femoral artery with a 5Fr prelude (Merit Medical Systems, Inc, UT, USA) sheath under ultrasound guidance. An angled Van Schie 2 catheter (Cook Medical, Inc) and 0.018 Glidewire® (Terumo; Somerset, New Jersey, USA) were used to access the left DCIA and a Progreat® 2.4Fr microcatheter (Terumo; Somerset, New Jersey, USA) used to cannulate the aneurysmal sac.

**Figure 3:**
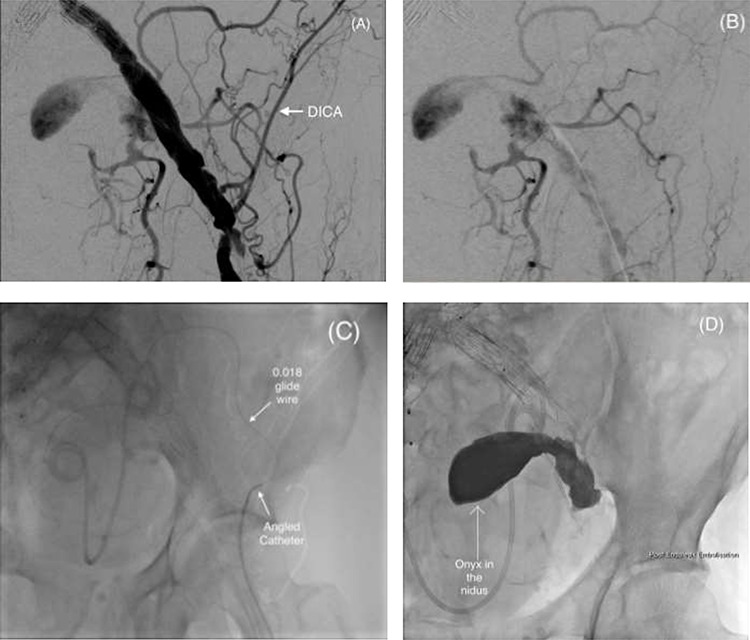
(**A** and **B**) Pre-embolization digital subtraction angiography (DSA) via a retrograde ipsilateral common femoral artery puncture demonstrating the left IIAA with inflow from the deep pelvic arteries including the DICA. (**C**) Microcatheter utilization to cannulate the aneurysm nidus with an angled catheter over an 0.018 hydrophilic Glidewire® to access the DICA. (**D**) Onyx™ successfully injected into the IIAA nidus.

A 21-ml total volume mixture of low and high viscosity ethylene-vinyl alcohol copolymer (EVOH), Onyx™ LES 18 and 34 respectively, was used for the successful embolization of the nidus (Fig. 3A–C). Completion angiogram demonstrated no further perfusion of the IIAA, and the patient was discharged home the same day. A follow-up of 90-day imaging demonstrated continued IIAA occlusion, with no sac expansion.

## DISCUSSION

Endoleaks, a complication unique to EVAR, remain the largest cause of reintervention. T2E are the result of retrograde blood flow from collateral vessels into the aneurysm sac, with the superior- or inferior-gluteal arteries more commonly associate with IIAA. [2, 5–6] Mostly, T2E are managed conservatively with spontaneous thrombosis and resolution is seen in 50% of the cases [1, 2].

In our case, the emergent nature of the EVAR and the patient’s tortuous iliac arterial anatomy precluded left IIA embolization prior to bifurcated device stent deployment. Subsequent persistent retrograde flow through the deep circumflex iliac artery (DICA) contributed to the IIAA development. The use of Onyx™ is a safe and effective modality for the treatment of T2E, however precise Onyx™ deposition remains unpredictable [7, 8]. Traditionally, for large endoleaks the use of coils as an adjunct to minimize the risk of distal embolization has been utilized with good technical success rates [9]. Available as two concentrations of EVOH (Onyx LES 18 and Onyx LES 34), understanding of the properties of each is paramount to maximizing their benefits. Onyx™ 18’s lower viscosity is desirable for very distal embolization, whilst Onyx™ 34’s higher viscosity makes it preferable for embolization of large sacs [9]. We advocate for the novel technique of mixing both high (Onyx™ 18) and low (Onyx™ 34) viscosity formulations at a ratio of 1:1 to maximize the properties of both polymers when attempting transarterial embolization of larger aneurysms. The non-adherent nature, minimal inflammatory effect on the vascular endothelium and progressive solidification characteristics of Onyx are advantageous [8].

## CONCLUSION

In conclusion, techniques to treat T2E continue to evolve with the accompanying rise of EVAR. Endoleaks resulting in IIAA are a technically challenging clinical dilemma often as a result of complicated anatomy. A range of percutaneous treatment methods are described in the literature with varying success. Onyx™ copolymer for the embolization of T2E is a suitable option, with increasing success rates dependent on user familiarity and experience.
